# Supplementary material for: Diversity within the adenovirus fiber knob hypervariable loops influences primary receptor interactions
Source: Nat Commun. 2019 Feb 14;10:741. doi: 10.1038/s41467-019-08599-y (PMC6376029; doi:10.1038/s41467-019-08599-y)
Supplement: Supplementary file 1 — Supplementary Information [file 41467_2019_8599_MOESM1_ESM.pdf]

Supplementary Information

Diversity within the adenovirus fiber knob hypervariable loops influences primary receptor interactions.

Baker *et al*.

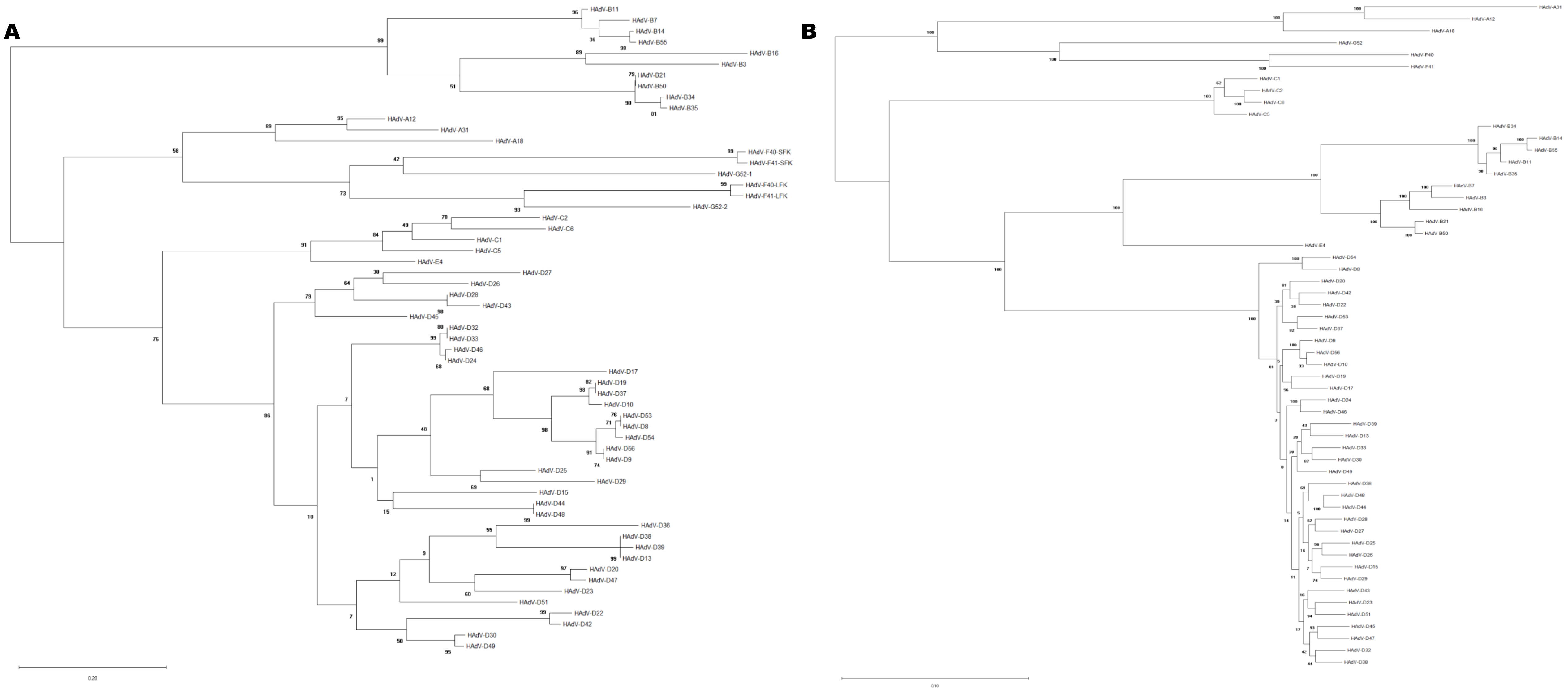

Supplementary Figure 1: Phylogenetic analysis of adenoviruses by whole genome and fiber-knob domain. Dendrograms showing uncondensed maximum likelihood trees (percentage confidence shown by numbers next to nodes) were generated from alignments of fiber-knob domain amino acid sequences of adenoviruses 1-56 (A) or whole genome NT sequences (B). While greater diversity can be seen compared to the condensed tree, many nodes are poorly supported.

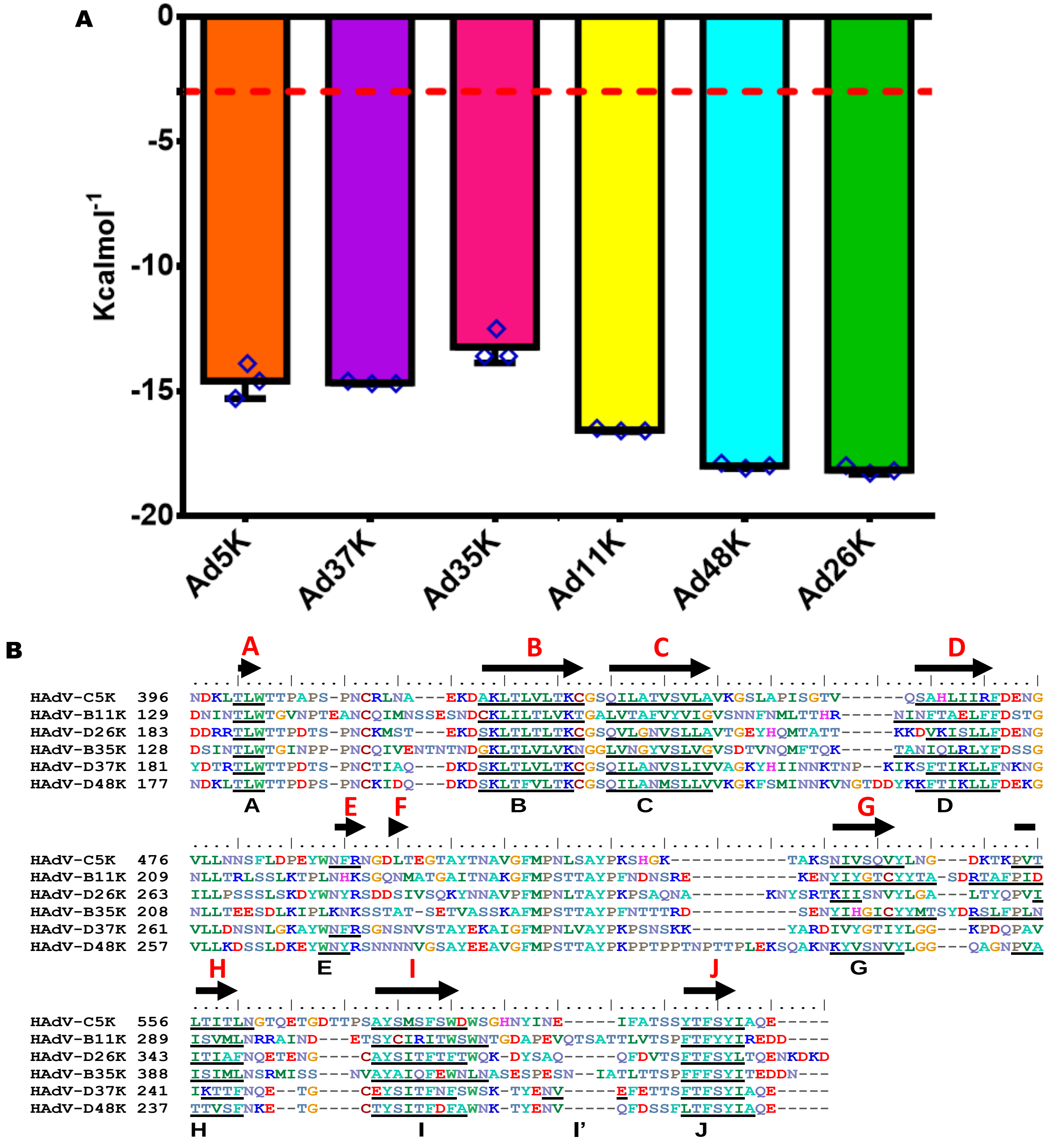

Supplementary Figure 2: HAdV-D26 and HAdV-D48 fiber-knob domains are predicted to form more stable trimers that HAdV-C5 fiber-knob but have similar overall topology. Interface energy calculations performed using PISA are shown on the bar chart with lower values indicating more stable interfaces (A). Amino acid sequence alignment of the 6 tested adenovirus fiber-knob domains is shown with the β-strand regions (underlined) (B). The arrows indicate the positions of the β-strands within the HAdV-C5K structure originally defined by Xia et al (1995), numbering indicates the amino acid position from the first amino acid of that species' fiber-knob protein. n=3, where each calculation is an independent fiber-knob: CAR interface, error bars indicate mean±SD.

Supplementary Figure 3: Analysis of polar contacts between HAdV-D26K and HAdV-D48K loops suggests an intricate network supporting specific loop conformations. The residues comprising the GH and IJ loops of HAdV-D26K (A) and HAdV-D48K (B) are shown diagrammatically. Numbers indicate the position of the loops' terminal residues in the fiber protein. A network of interloop polar interactions is shown by solid lines (one polar bond), and dashed lines (two polar bonds), residues forming part of a helical motif are shaded in blue. The same analysis is seen for the HI, DG, IJ, and CD loops of HAdV-D26K (C) and HAdV-D48K (D).

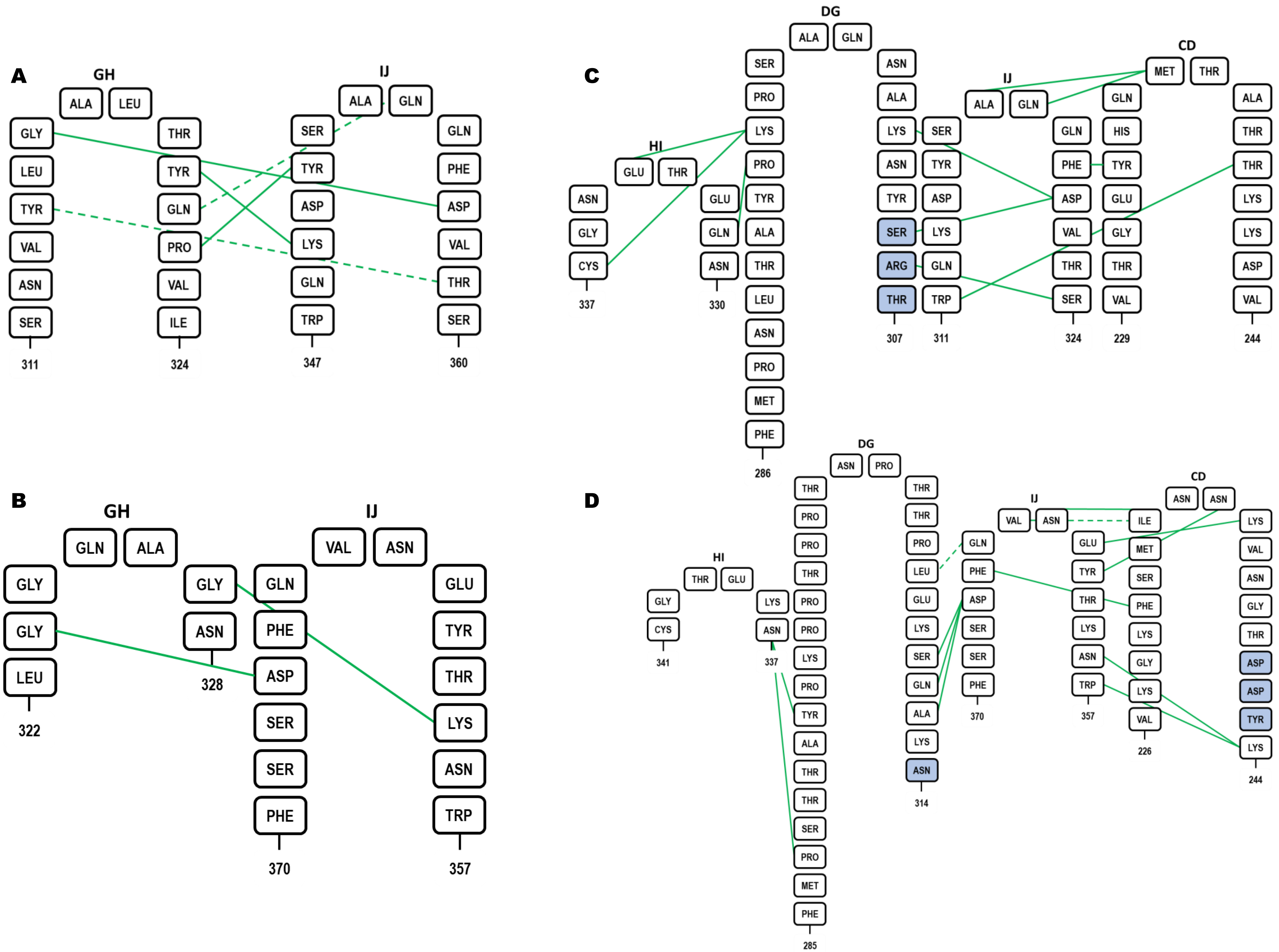

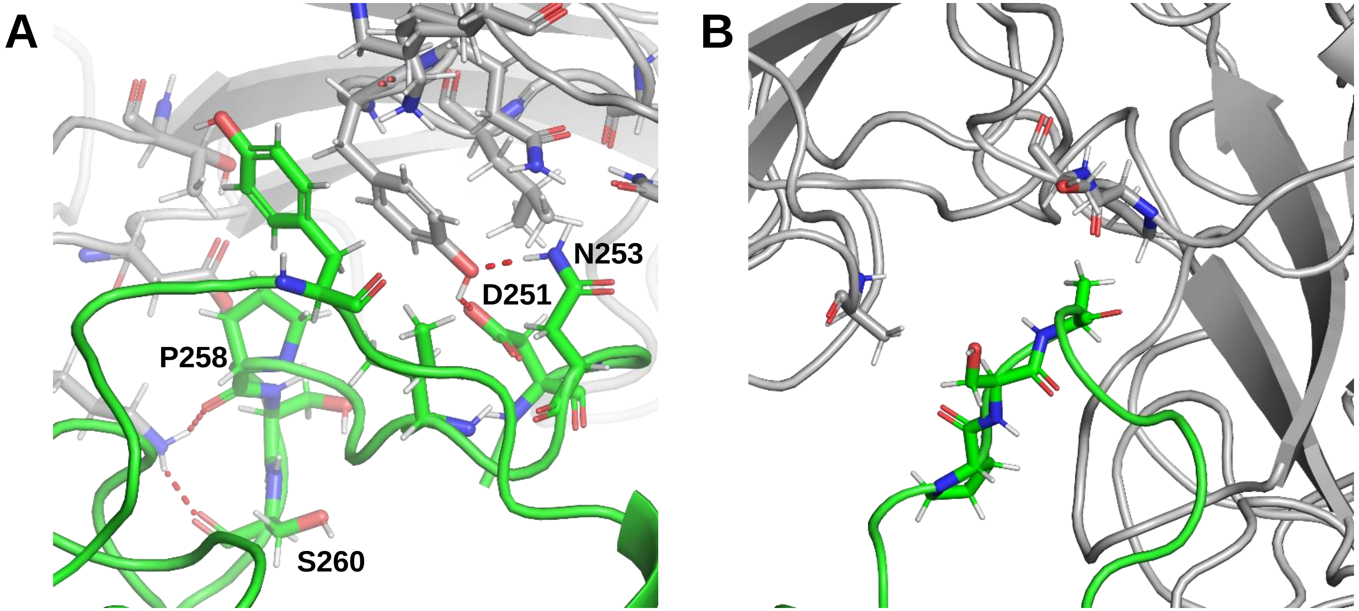

Supplementary Figure 4: Crystal packing contacts formed by the DG-loop of HAdV-D26K. Sticks show residues forming crystallographic contacts between the DG-loop of HAdV-D26K (Green) and monomers outside of the biological trimer (Grey). Residues forming polar bonds (Red dashes) are labelled, all other residues are forming Van Der Waals (VDW) contacts at a radius of 3.5Å. Red and blue atoms are oxygen and nitrogen, respectively.

```
HAdV-C5     FMPNLSAYPKSHGK-----------TAKSNIVSQV
HAdV-D45    FMPNLVAYPRPNTPDS-------KIYARSKIVGNV
HAdV-D28    FMPNITAYKP---VNS-------KSYARSHIFGNV
HAdV-D43    FMPNITAYKP---TNS-------KSYARSVIFGNV
HAdV-D26    FMPNLTAYPKPSAQNA-------KNYSRTKIISNV
HAdV-D27    FMPNLVAYPKPTSADA-------KNYSRSKIISNV
HAdV-D25    FMPNLAAYPKSTTTQS-------KLYARNTIFGNI
HAdV-D29    FMPNLLAYAKATTDQS-------KIYARNTIYGNI
HAdV-D17    FMPNLVAYPKPTT-GS-------KKYARDIVYGNI
HAdV-D10    FMPNLVAYPKPSN--S-------KKYARDIVYGTI
HAdV-D19    FMPNLVAYPKPSN--S-------KKYARDIVYGTI
HAdV-D37    FMPNLVAYPKPSN--S-------KKYARDIVYGTI
HAdV-D8     FMPNLVAYPKPTT-GS-------KKYARDIVYGNI
HAdV-D53    FMPNLVAYPKPTT-GS-------KKYARDIVYGNI
HAdV-D54    FMPNLVAYPKPTT-GS-------KKYARDIVYGNI
HAdV-D9     FMPNLVAYPKPTA-GS-------KKYARDIVYGNI
HAdV-D56    FMPNLVAYPKPTA-GS-------KKYARDIVYGNI
HAdV-D13    FMPNLKAYPKPTKTASDK-AENKISSAKNKIVSNF
HAdV-D38    FMPNLKAYPKPTKTASDK-AENKISSAKNKIVSNF
HAdV-D39    FMPNLKAYPKPTKTASDK-AENKVSSAKNKIVSNF
HAdV-D36    FMPSTTAYPKPTNNTSTD-PDKKVSQGKNKIVSNI
HAdV-D51    FMPNLKAYPKNTTTSSTN-PDDKISAGKKNIVSNV
HAdV-D23    FMPNLKAYPNPTTSTTNP-STDKKSNGKNAIVSNV
HAdV-D20    FMPNLKAYPKP--STVLP-STDKNSNGKNTIVSNL
HAdV-D47    FMPNLKAYPNPKTSTVLP-STDKKSNGKNTIVSNL
HAdV-D32    FMPNIKAYPKPTTDTSA-KPEDKKSAAKRYIVSNV
HAdV-D33    FMPNIKAYPKPTTDTSA-KPEDKKSAAKRYIVSNV
HAdV-D24    FMPNIKAYPKPTTDTSA-KPEDKKSAAKRYIVSNV
HAdV-D46    FMPNIKAYPKPSTDTSA-KPEDKKSAAKRYIVSNV
HAdV-D22    FMPNTTAYPKIIDSTTNP--ADKKSSAKKIIVGNV
HAdV-D42    FMPNTTAYPKIINSTTDP--ENKKSSAKKTIVGNV
HAdV-D15    FMPSKTAYPKQTKPT-----NKEISQAKNKIVSNV
HAdV-D44    FMPSTTAYPKPPTPPTNPTTPLEKSQAKNKYVSNV
HAdV-D48    FMPSTTAYPKPPTPPTNPTTPLEKSQAKNKYVSNV
HAdV-D30    FMPNSTAYPKIINNGTAN-PEDKKSAAKKTIVTNV
HAdV-D49    FMPNSTAYPKIINNGTAN-PEDKKSAAKKTIVTNV
```

Supplementary Figure 5: Species D adenoviruses possess a range of DG loop sequences varying in both length and sequence. The DG loops of all species D adenoviruses are shown aligned, using clustal omega, to the same region in HAdV-C5 (species C).

```
             ....|....| ....|....| ....|....| ....|....| ....|....| ....|....| ....|....|
HAdV-D37K    TLWTTPDTSP NCTIAQDKDS KLTLVLTKCG SQILANVSLI VVAGKYHIIN NKTNP--KIK SFTIKLLFNK
HAdV-G52SFK  TLWTPPTSNP NCTVYTESDS LLSLCLTKCG AHVLGSVSLT GVAGTMTNMA E--------T SLAIEFTFDD
CAV-2        TLWTGPGPSI NGFINDTPVI RCFICLTRDS NLVTVNASFV GE-GGYRIVS PT------QS QFSLIMEFDQ
HAdV-D26K    TLWTTPDTSP NCKMSTEKDS KLTLTLTKCG SQVLGNVSLL AVTGEYHQMT ATT-----KK DVKISLLFDE
HAdV-D48K    TLWTTPDPSP NCKIDQDKDS KLTFVLTKCG SQILANMSLL VVKGKFSMIN NKVNGTDDYK KFTIKLLFDE

             ....|....| ....|....| ....|....| ....|....| ....|....| ....|....| ....|....|
HAdV-D37K    NGVLLDNSNL GK-AYWNFRS GNSN--VSTA YEKAIGFMPN LVAYPKPSNS KK-------- -YARDIVYGT
HAdV-G52SFK  TGKLLHSPL- VN-NTFSIRQ GDSP--ASNP TYNALAFMPN STLYARGGSG ---------- -EPRNNYYVQ
CAV-2        FGQLMSTGNI NSTTTWGEKP WGNNTVQPRP SHTWKLCMPN REVYSTPAAT ISR------- ----C-GLDS
HAdV-D26K    NGILLPSSSL SK-DYWNYRS DDSI--VSQK YNNAVPFMPN LTAYPKPSAQ NA-------K NYSRTKIISN
HAdV-D48K    KGVLLKDSSL DK-EYWNYRS NNNN--VGSA YEEAVGFMPS TTAYPKPPTP PTNPTTPLEK SQAKNKYVSN

             ....|....| ....|....| ....|....| ....|....| ....|....| ....|....| ...
HAdV-D37K    IYLGGKPDQP AVIKTTFNQE --TGCEYSIT FNFS-WSKTY ENVEFETTSF TFSYIAQE-- ---
HAdV-G52SFK  TYLRGNVQRP ITLTVTFNSA A---TGYSL- -SFK-WTAVV -REKFAAPAT SFCYITEQ-- ---
CAV-2        IAVDGAPSRS IDCMLIINKP K-GVATYTLT FRFLNFNRLS GGTLFKTDVL TFTYVGENQ- ---
HAdV-D26K    VYLGALTYQP VIITIAFNQE TENGCAYSIT FTFT-WQKDY SAQQFDVTSF TFSYLTQENK DKD
HAdV-D48K    VYLGGQAGNP VATTVSFNKE --TGCTYSIT FDFA-WNKTY ENVQFDSSFL TFSYIAQE-- ---
```

Supplementary Figure 6: Previously described sialic acid using adenovirus fiber-knob sequences aligned to HAdV-D26 and HAdV-D48. The fiber-knob domains, defined as the conserved TLW hinge motif to the C-terminus, of the known sialic acid utilising HAdV-D37, HAdV-G52 short fiber-knob, and CAV-2, aligned to the fiber-knob domains of HAdV-D26K and HAdV-D48K by clustal omega. Residues highlighted in red have been described as direct contacts between HAdV-D37K and sialic acid. Those in yellow form water bridges to sialic acid, from HAdV-D37K. Those highlighted in blue have been shown to be important to the charge dependent HAdV-G52K to poly-sialic acid interaction, and those in green shown to be involved in CAV-2's interaction with sialic acid.

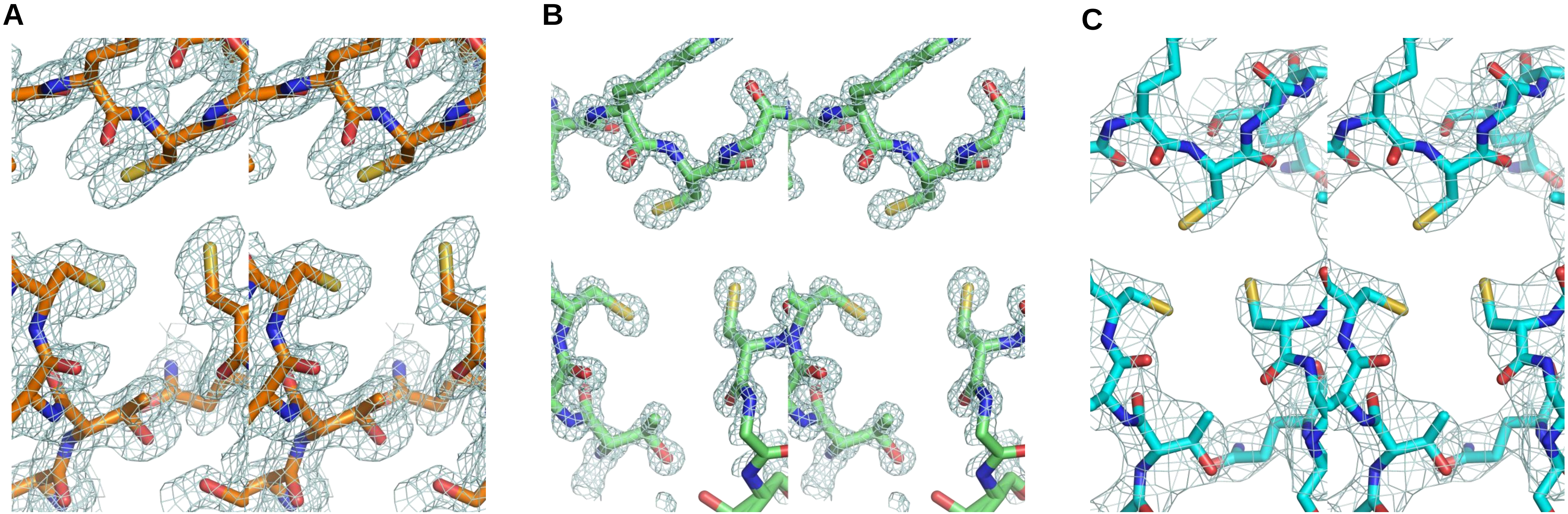

Supplementary Figure 7: Stereo Images of a representative region of model and map for crystallographic structures determined in this study. Stereo images of HAdV-C5K (PDB 6HCN) (**A**), HAdV-D26K (PDB 6FJN) (**B**), and HAdV-D48K (PDB 6FJQ) (**C**) are shown coloured in orange, green, and cyan, respectively. Oxygen, nitrogen, and sulphur atoms are coloured in red, blue, and yellow, respectively. The displayed 2Fo-Fc map is contoured at 1.5σ for HAdV-D26K, and at 1.0σ for HAdV-C5K and HAdV-D48K.

Supplementary Table 1: Binding energy for each loop of HAdV-D26 and HAdV-D48 resulting from crystal packing contacts, outside of the biological trimer, and the number of polar bonds formed.

	**HAdV-D26K**		**HAdV-D48K**	
**Loop Identity**	**Total Binding Energy (KcalMol^{-1})**	**Number of Polar Contacts**	**Total Binding Energy (KcalMol^{-1})**	**Number of Polar Contacts**
**AB**	-0.6	0	N/A	0
**BC**	N/A	0	N/A	0
**CD**	-0.3	2	-2.6	3
**DG**	-6.5	4	-2.4	0
**GH**	-3.0	2	N/A	0
**HI**	N/A	0	N/A	0
**IJ**	N/A	0	-0.4	0

Supplementary Table 2: Data collection and refinement statistics for new HAdV-C5, HAdV-D26, and HAdV-D48 Fiber-knob protein crystal structures deposited in the PDB.

**PDB Entry**	**6FJN**	**6HCN**	**6FJQ**
Diamond Beamline	I04	I24	I04
Date	2017-05-12	2018-01-26	2017-05-12
Wavelength	0.9795	0.96859	0.9795
Crystallisation Conditions	0.1 M MMT 25% PEGA 1500	0.1 M MMT, 25% PEG 1500	0.1 M Bis-Tris-propane, 20% PEG 3350, 0.2M NaNO3
pH	6.0	7.0	6.5
a,*b*,*c* (Å)	86.01,86.01,86.01	102.16,102.44,77.0	145.18,145.18,145.18
$\alpha=\beta=\gamma$ (°)	90.0	90.0	90.0
Space group	$P\ 2_1\ 3$	$P\ 2_1\ 2_1\ 2$	$P\ 4_3\ 3\ 2$
Resolution (Å)	0.97-60.82	1.49-61.56	2.91-83.82
Outer shell	0.97-1.00	1.49-1.53	2.91-2.99
R-merge (%)	4.3 (74.5)	13.4 (183.8)	12.5 (302.6)
R-meas (%)	4.5 (97.5)	15.9 (218.3)	12.7 (306.3)
CC1/2	1.00 (0.427)	0.983 (0.565)	1.00 (0.705)
I / σ(I)	27.3 (0.7)	7.1 (0.7)	22.2 (1.7)
Completeness (%)	94.9 (43.9)	99.8 (99.9)	100.0 (100.0)
Multiplicity	16.7 (1.6)	6.6 (6.3)	41.2 (41.4)
Total Measurements	1,978,768 (6,429)	876,648 (60,950)	496,751 (5,136)
Unique Reflections	118,603 (4,055)	131,951 (9,638)	12,061 (877)
Wilson B-factor($Å^2$)	8.2	18.3	74.5
R-work reflections	112,612	125,479	11,371
R-free reflections	5,879	6,388	572
R-work/R-free (%)	18.2 / 19.5	21.1/23.3	20.1 / 29.1
Bond lengths (Å)	0.025	0.011	0.019
Bond Angles (°)	2.339	1.534	2.293
[1]Coordinate error	0.020	0.087	0.370
Mean B value ($Å^2$)	17.6	30.6	84.9
Favoured/allowed/ Outliers	138 / 7 / 1	133 / 10 / 1	350 / 28 / 7
%	94.5 / 4.8 / 0.7	92.4 / 6.9 / 0.7	90.7 / 7.5 / 1.8

* One crystal was used for determining each structure.
* Figures in brackets refer to outer resolution shell, where applicable.
[1] Coordinate Estimated Standard Uncertainty in (Å), calculated based on maximum likelihood statistics.

Buffers:
- MMT: DL-Malic acid, MES monohydrate, Tris: pH 4.0-9.0
- SPG: Succinic acid, Sodium phosphate monobasic monohydrate, Glycine: pH 4.0-10.0
