# Supplementary material for: Diversity within the adenovirus fiber knob hypervariable loops influences primary receptor interactions
Source: Nat Commun. 2019 Feb 14;10:741. doi: 10.1038/s41467-019-08599-y (PMC6376029; doi:10.1038/s41467-019-08599-y)
Supplement: Supplementary file 3 — Reporting Summary [file 41467_2019_8599_MOESM3_ESM.pdf]

nature research

Corresponding author(s): *Double-blind peer review submissions: write DBPR and your manuscript number here instead of author names.*

Last updated by author(s): *YYYY-MM-DD*

Reporting Summary

Nature Research wishes to improve the reproducibility of the work that we publish. This form provides structure for consistency and transparency in reporting. For further information on Nature Research policies, see Authors & Referees and the Editorial Policy Checklist.

Statistics

For all statistical analyses, confirm that the following items are present in the figure legend, table legend, main text, or Methods section.

n/a	Confirmed	
☐	☒	The exact sample size (n) for each experimental group/condition, given as a discrete number and unit of measurement
☐	☒	A statement on whether measurements were taken from distinct samples or whether the same sample was measured repeatedly
☒	☐	The statistical test(s) used AND whether they are one- or two-sided<br>*Only common tests should be described solely by name; describe more complex techniques in the Methods section.*
☒	☐	A description of all covariates tested
☐	☒	A description of any assumptions or corrections, such as tests of normality and adjustment for multiple comparisons
☐	☒	A full description of the statistical parameters including central tendency (e.g. means) or other basic estimates (e.g. regression coefficient) AND variation (e.g. standard deviation) or associated estimates of uncertainty (e.g. confidence intervals)
☒	☐	For null hypothesis testing, the test statistic (e.g. F, t, r) with confidence intervals, effect sizes, degrees of freedom and P value noted<br>*Give P values as exact values whenever suitable.*
☒	☐	For Bayesian analysis, information on the choice of priors and Markov chain Monte Carlo settings
☒	☐	For hierarchical and complex designs, identification of the appropriate level for tests and full reporting of outcomes
☐	☒	Estimates of effect sizes (e.g. Cohen's d, Pearson's r), indicating how they were calculated

Our web collection on statistics for biologists contains articles on many of the points above.

Software and code

Policy information about availability of computer code

Data collection	Diffraction data was analysed and reduced using XDS, XIA2, DIALS, and Autoproc using the version on the Diamond Light Source pipeline.<br>Structure determination and refinemtn was performed using the components of the CCP4i package, v7.0.066.<br>Wincoot v0.8.9.1 was used to adjust models.<br>Interface energy calculations were performed using QtPISA v2.1.0.
Data analysis	Models were analysed using PyMOL v2.0.4. Flow cytometry data was analysed using FlowJo VX.0.7. Graphs were generated in GraphPad Prism v6.0.1

For manuscripts utilizing custom algorithms or software that are central to the research but not yet described in published literature, software must be made available to editors/reviewers. We strongly encourage code deposition in a community repository (e.g. GitHub). See the Nature Research guidelines for submitting code & software for further information.

Data

Policy information about availability of data

All manuscripts must include a data availability statement. This statement should provide the following information, where applicable:

- Accession codes, unique identifiers, or web links for publicly available datasets
- A list of figures that have associated raw data
- A description of any restrictions on data availability

Data Availability Statement.

Macromolecular structures generated during this study have been deposited in wwPDB (worldwide Protein Data Bank; https://www.wwpdb.org/), and have PDB ID's 6FJN, 6HCN, and 6FJQ. PDB ID's for macromolecular structures utilised, but not generated in the course of this study, are as follows: HAdV-B11K in complex with CD46, PDB 3O8E. HAdV-D37K in complex with CAR-D1, PDB 2J12. HAdV-B35K, PDB 2QLK.

Genomic sequences from which fiber-knob domain sequences were determined, which have been used in phylogenetic analysis, have the following NCBI accession

numbers: AC_000017|AF532578|X73487|AY803294|AB562586|AY601636|AF108105|GU191019|JQ326209|AC_000007|JN226749|KF528688|FJ404771|JN226750|JN226751|JN226752|EF153474|JN226753|FJ824826|JN226754|JN226755|AM749299|JN226756|JN226758|AY737797|AC_000019|GQ384080|JN226759|JN226760|KU162869|DQ315364|JN226761|JN226762|JN226763|JN226764|AY875648|JN226757|EF153473|DQ393829|AC_000008|AY737798|JN226765|DQ923122|AB605243|NC_012959|FJ643676|HM770721|HQ413315|AC_000018|DQ086466|JN226746|JN226747|AB448776|AB448767|AJ854486|KF006344|

All other data pertaining to this manuscript are available from the authors upon request.

Field-specific reporting

Please select the one below that is the best fit for your research. If you are not sure, read the appropriate sections before making your selection.

☒ Life sciences ☐ Behavioural & social sciences ☐ Ecological, evolutionary & environmental sciences

For a reference copy of the document with all sections, see nature.com/documents/nr-reporting-summary-flat.pdf

Life sciences study design

All studies must disclose on these points even when the disclosure is negative.

Sample size	IC50 Experiments had n=3 biological replicates. Interface energy calculations had n=3 where each replicate was a new calculation performed on an independent protein-protein interface. Phylogeny studies utilized on sequence per adenovirus serotype investigated, the accession numbers for the sequences utilized are in the data availability statement.
Data exclusions	No data was excluded.
Replication	All replication attempts were sucessful
Randomization	No parts of this study involved the selection of random participants, thus this is not applicable.
Blinding	There was no group selection, thus blinding is not applicable

Reporting for specific materials, systems and methods

We require information from authors about some types of materials, experimental systems and methods used in many studies. Here, indicate whether each material, system or method listed is relevant to your study. If you are not sure if a list item applies to your research, read the appropriate section before selecting a response.

Materials & experimental systems

n/a	Involved in the study	
☐	☒	Antibodies
☐	☒	Eukaryotic cell lines
☒	☐	Palaeontology
☒	☐	Animals and other organisms
☒	☐	Human research participants
☒	☐	Clinical data

Methods

n/a	Involved in the study	
☒	☐	ChIP-seq
☐	☒	Flow cytometry
☒	☐	MRI-based neuroimaging

Antibodies

Antibodies used	Alexa-647 labelled goat anti-mouse F(ab')2. ThermoFisher - #A-21237<br>Anti-CAR Antibody, clone RmcB - Millipore - 05-644<br>CD46 Monoclonal Antibody (MEM-258) - Abcam - Ab789
Validation	Anti-CAR antibody RmcB is validated as described at the following URL: http://www.merckmillipore.com/GB/en/product/Anti-CAR-Antibody-clone-RmcB,MM_NF-05-644?bd=1#documentation<br>Anti-CD46 antibody MEM-258 is validated as described at the following URL: https://www.thermofisher.com/antibody/product/CD46-Antibody-clone-MEM-258-Monoclonal/MA1-82140

Eukaryotic cell lines

Policy information about cell lines

Cell line source(s)	Cell lines, CHO-CAR and CHO-BC1, were a kind gift from George Santis, original cell line derivations are references in the methods

Authentication	CHO-CAR and CHO-BC1 cells were validated for expression of CAR and CD46 receptor by flow cytometric analysis
Mycoplasma contamination	Cell lines tested negative for mycoplasma contamination
Commonly misidentified lines (See ICLAC register)	N/A

Flow Cytometry

Plots

Confirm that:

☐ The axis labels state the marker and fluorochrome used (e.g. CD4-FITC).

☐ The axis scales are clearly visible. Include numbers along axes only for bottom left plot of group (a 'group' is an analysis of identical markers).

☐ All plots are contour plots with outliers or pseudocolor plots.

☐ A numerical value for number of cells or percentage (with statistics) is provided.

Methodology

Sample preparation	*Describe the sample preparation, detailing the biological source of the cells and any tissue processing steps used.*
Instrument	*Identify the instrument used for data collection, specifying make and model number.*
Software	*Describe the software used to collect and analyze the flow cytometry data. For custom code that has been deposited into a community repository, provide accession details.*
Cell population abundance	*Describe the abundance of the relevant cell populations within post-sort fractions, providing details on the purity of the samples and how it was determined.*
Gating strategy	*Describe the gating strategy used for all relevant experiments, specifying the preliminary FSC/SSC gates of the starting cell population, indicating where boundaries between "positive" and "negative" staining cell populations are defined.*

☐ Tick this box to confirm that a figure exemplifying the gating strategy is provided in the Supplementary Information.
